# Measuring Cardiovascular Disease Risk Perception: Translation and Validation of the Indonesian ABCD Risk Questionnaire

**DOI:** 10.1155/2024/7526455

**Published:** 2024-09-03

**Authors:** Septa Meriana Lumbantoruan, Deby Kristiani Uligraff, Eva Berthy Tallutondok

**Affiliations:** ^1^ Faculty of Nursing Universitas Pelita Harapan, Tangerang, Indonesia; ^2^ Department of Nursing Tarumanagara Institute, Jakarta, Indonesia

## Abstract

**Background:**

Cardiovascular diseases (CVDs) are still increasing worldwide contributing to increasing death worldwide. To test CVDs' awareness, the Attitude and Belief about Cardiovascular Disease (ABCD) questionnaire was developed. However, this questionnaire is not available in Indonesia language.

**Methods:**

The original questionnaire was translated in both directions forward and backward. The process is then continued with a content validity index created by three experts. The exploratory factor analysis (EFA) and confirmatory factor analysis (CFA) then determine the factors that support the translated questionnaire. The splitting sample method was applied in both factor analyses. Internal consistency testing of 18 items was performed on 236 samples.

**Result:**

The validity of the entire questionnaire subscale was satisfactory. Three retained factors were supported by the EFA and CFA, namely, risk perception, perceived benefit, and healthy eating intention. The internal consistency was acceptable based on Cronbach alpha and ordinal alpha. The Indonesian version of ABCD questionnaire was statistically valid and reliable to be used.

**Conclusion:**

The Indonesian version of the ABCD questionnaire is a valid questionnaire to access the attitude and belief of CVDs in Indonesia.

## 1. Introduction

Globally, 17.9 million people died in 2019 due to cardiovascular diseases (CVDs) [1]. This number rose to 19.1 million by 2020 and will continue to increase annually [2]. The number of CVDs is highest in Asia and Africa, with disability-adjusted life years (DALYs) ranging from 6.154 to 20.998 per 100,000 people [3]. A similar number of DALYs are present in Indonesia [3]. Low- and middle-income countries account for about 75% of all cardiovascular disease cases worldwide [1]. In 2018, 4.2 million people in Indonesia, a low- and middle-income country, were diagnosed with CVDs [4]. The rising prevalence of cardiovascular disease makes it the leading cause of death worldwide [1].

A growing number of CVDs are caused by risk factors [3]. High blood pressure, dietary risk, high LDL, tobacco, high body mass index, high blood glucose, kidney dysfunction, and many other risk factors lead to CVDs [3]. Reducing risk factors is essential to prevent CVDs [5]. Health behaviors that minimize risk factors include increasing physical exercise, eating a healthy diet, maintaining a healthy body mass index, quitting smoking, and managing stress [5]. Health beliefs affect health behavior based on someone's knowledge and perception [6]. Sufficient knowledge of CVDs motivates people to seek healthy behaviors [7].

Instruments to measure the knowledge of CVDs are still lacking. A narrative review reported there were twelve instruments to measure the knowledge of cardiovascular risk; however, only four of them measure specifically about CVDs [8]. The National Health Service of the United Kingdom previously developed the Attitude and Beliefs about Cardiovascular Disease (ABCD) Risk Questionnaire to measure attitudes and beliefs about cardiovascular disease [9]. This questionnaire has been translated into many different languages around the world [10–14]. A reliable and valid questionnaire is critical to measuring knowledge and perceptions of CVDs. Indonesia, as a major Southeast Asian country, contributes to a high incidence of CVDs [15]. Therefore, it is necessary to translate the ABCD questionnaire into the Indonesian version.

## 2. Materials and Methods

### 2.1. Study Design and Period of the Study

This study was an instrumental questionnaire validation study which conducted in Indonesia from May 2023 to June 2023.

### 2.2. Study Setting and Study Populations

The internet platform was utilized to recruit samples via convenience sampling. Information about this study was distributed through social media platforms such as Facebook, Instagram, and WhatsApp because these platforms are popular and commonly used in Indonesia.

### 2.3. Eligibility

An inclusion criterion was being an adult (>18 years old) based on the law of Indonesia and being able to communicate well in Indonesian language. People with mental disorders are an exclusion criterion.

### 2.4. Sample Size Determination and the Sampling Procedure

The sample size's power was determined using *G* power post hoc analysis. The power was 99% for an exact test with two tails and a correlation bivariate normal model (alpha error was 0.05 and sample size is 236). This study included a total sampling of respondents. The splitting sample method was utilized in factor analysis [16], with 118 samples in exploratory factor analysis and 118 samples in confirmatory factor analysis. The previous recommendation said that at least 50 samples are required to do factor analysis [17].

### 2.5. Data Collection Tool

A self-administered tool was utilized to collect information from the respondents. The tools include demographic data, smoking habits, body weight and height, exercise activities, and an ABCD questionnaire.

### 2.6. Instruments

#### 2.6.1. The Original ABCD Questionnaire

In 2017, an ABCD questionnaire was developed to assess individuals' knowledge, perceived risks, and benefits of CVDs [9]. It consists of 26 items divided into the following four dimensions: knowledge (8 items), perceived risk of heart attack/stroke (8 items), perceived benefits and intentions to change (7 items), and healthy eating intentions (3 items). The first dimension is on a Guttman scale, while the others are on the Likert scale (1–4). Exception in scoring will be applied to the following four items: item number 8, 15, 21, and 28. Item number 8 will be scored “1” if the respondents choose “wrong;” furthermore, items number 15, 21, and 26 required unfavorable answer to get a higher score. The minimum score was 18, and the maximum score was 80 for the overall scale. The higher the score on the ABCD Risk Questionnaire, the greater the health belief in CVDs' perception [9].

### 2.7. Data Collection Procedure

From May 2023 to June 2023, information about this study was disseminated via social media platforms such as Facebook, Instagram, and WhatsApp. Four research assistants were employed in this study to collect the data. They were nursing students in their four-year study and had been taught about research courses. The explanation of the study, respondent recruitment, informed consent, and data collection were given to the research assistant. For the respondents, it took 15 minutes to fill in the questionnaire.

### 2.8. Data Analysis

Internal validity was analyzed using the content validity index (CVI), and a value of 0.78 or higher is considered excellent content validity [18]. Statistical analysis was done with statistical software for data science (STATA) version 18. The structural analysis of the questionnaire was assessed using factor analysis. EFA was employed to identify the latent construct underlining a set of the ABCD's variables. The EFA assumptions were tested (sample size, distribution, collinearity, and linearity) [17, 19], and the including the data were continuous variables, sample size was over than 100, and the data were in normal distribution (*p* > 0.05) based on Kolmogorov–Smirnov and Shapiro–Wilk tests. The multicollinearity was checked using variance inflation factor (VIF), which ranges from 1 to 6 (items 10 and 11 have VIF score 6). If the VIF is less than 10, no multicollinearity. The linearity was observed using Q-Q plot and, the data show a linear relationship between observed variables, with no outliers. The methods of extraction used in this study was principal axis factoring due to the data was not distributed normally using Kolmogorov–Smirnov and Shapiro–Wilk test (*p* < 0.05). For the rotation methods, we use orthogonal, especially varimax rotation to minimize cross loading. The selection criteria of number of factors to be retained is based on the eigenvalue >1 rule, scree plot, variance extracted, and Barlett's chi-square test [20]. CFA was done to prove the structure of ABCD Risk Questionnaire using the new data from Indonesia. The model fit of the CFA model was assessed including X^2^/df, comparative fit index (CFI), Tucker–Lewis's index (TLI), and root mean square error of approximation (RMSEA) and standardized root mean squared error (SRMR) [21]. The reliability of the ABCD questionnaire was analyzed by test-retest reliability and internal consistency coefficient using Cronbach's alpha and Ordinal alpha. All variables were described in a univariate analysis.

### 2.9. Ethical Consideration

The ethical approval was acquired from the Research Ethics Committee of Sekolah Tinggi Ilmu Kesehatan Bani Saleh under the number EC.228/KEPK/STKBS/XI/20di22. Respondents who participated in this study were willing to provide informed consent via an online form. Respondents were asked for their initials without being asked for their email addresses to maintain anonymity and confidentiality. Furthermore, only the principal investigator accessed the link to the questionnaire.

## 3. Results

### 3.1. Translation and Adaptation of the Indonesian ABCD Questionnaire

Mrs. Woringer, the original author, granted permission to use the ABCD Risk Questionnaire. The World Health Organization (WHO) translation process was used, which included forward translation, reverse translation, an expert panel, pretesting, and the final version [22]. Forward translation was accomplished by a medical professional with over 15 years of experience pursuing a doctoral degree at the University of Auckland. A medical specialist translator did the backward translation with a doctoral degree from the University of North Texas and is currently working as a postdoctoral scholar at the Carol Nese College of Nursing at Pennsylvania State University. The expert panel had three experts, namely, a cardiologist from a government hospital, a nursing professor with a doctoral degree and an interest in cardiovascular research, and a nurse with nine years of hospital experience and three years of experience in the intensive coronary care unit. Following the discussion, a comprehensive ABCD questionnaire in Indonesia was developed. A pretest was carried out on 40 respondents to assess validity and reliability. The final step was analyzed and changed before the Indonesian version was obtained.

### 3.2. Sociodemographic Characteristics

The analysis was conducted with data from 236 samples who responded to the adapted and translated questionnaire. The respondents ranged from 18 to 44 years old, with a mean of 26.41 (SD 5.252). The respondents were predominantly female (*n* = 145, 61.4%). More than half of the respondents attended university (*n* = 160, 67.8%), followed by high school and secondary education (31.8% and 0.4%). The highest number of respondents are Christian protestants (*n* = 137, 58.1%), followed by Muslims (*n* = 75, 31.8%), Catholics (*n* = 17, 7.2%), Buddhas (*n* = 5, 2.1%), and Confucians (*n* = 2, 0.8%). Almost half of the total respondents are working for private companies (*n* = 116, 49.2%), followed by students (*n* = 54, 22.9%), unemployed (including full-time moms, *n* = 22, 9.3%), self-employed (*n* = 17, 7.2%), government employees (*n* = 16, 6.8%), and freelancers (*n* = 11, 4.7%). The distribution of monthly income showed that 35.2% (*n* = 83) of the respondents earned more than IDR 5.000.000, 25% (*n* = 59) had no income because they were students, 18.2% (*n* = 43) earned IDR 3.000.000–5.000.000, 14.4% (*n* = 34) earned IDR 1.000.000–3.000.000, and 7.2% (*n* = 17) earned less than IDR 1.000.000. The sociodemographic characteristics of respondents are listed in Table 1. Furthermore, the overall result of ABCD-I revealed that the scores ranged from 34 to 76 (the maximum score is 80) with a mean of 55.24 (SD = 5.837). All domains (knowledge, perceived risks, perceived benefits, and intention to change) exhibited more than 50% of the total marks in each band (Table 1).

### 3.3. The Validity of the ABCD-Indonesian Version

#### 3.3.1. Content Validity

As the World Health Organization suggested, the forward-backward translation was conducted before translating the ABCD Risk Questionnaire into the Indonesian language-translated version (hereafter ABCD-I) without compromising reliability and validity. The items were distributed into the following four domains: knowledge (8 items), perceived risk (8 items), perceived benefits (7 items), and intention to change (3 items). After getting the questionnaire translated, the expert panel process was conducted. Moreover, the expert panelists analyzed item by item and suggested revisions to make the item concise and understandable. The expert and team made revisions by considering several slight changes, such as adding prefixes and suffixes, to create sense-making sentences in the Indonesian language version.

Three experts were invited to evaluate the CVI of the ABCD-I Risk Questionnaire. The CVI was evaluated with the item content validity index (I-CVI), Scale CVI (S-CVI)/Ave, and S-CVI/UA. The I-CVI score was between 0.67 and 1.00, whereas the SCI/Ave and S-CVI/UA were 0.94 and 0.81, respectively. A CVI of ≥0.80 is recommended to prove that the determined item and questionnaire are clear, homogenous, and relevant. The expert agreed that all the items translated from the original questionnaire were preserved because they were considered essential, appropriate, and interlinked.

#### 3.3.2. Structural Validity

The structural validity of the ABCD Risk Questionnaire was evaluated to reflect the dimensionality of the items [23]. In the present study, EFA was utilized to investigate the underlying factors, whereas CFA was employed to verify the variable's factor structure. Both EFA and CFA were effective statistical procedures for ensuring the validity of the ABCD Risk Questionnaire. Before undertaking EFA and CFA, we evaluated the monotonicity and scalability of items using a Mokken scaling analysis (MSA). The result of both data showed *H* ≥ 0.4 reflects a moderate coefficient of scalability (H). All items in the ABCD-I questionnaire in EFA data were considered to be scalable (ranged between 0.485 and 0.872), and in CFA data, they also reflected to be scalable (ranged between 0.409 and 0.910).

The EFA is needed to explore the factorial structure of the ABCD Risk Questionnaire (Indonesia language version). The EFA was conducted using the principal axis factoring method of extraction with the varimax rotation method. Collected data deemed suitable to proceed into factor analysis based on the (1) Kaiser–Meyer–Olkin (KMO) measure, which indicated the compactness of correlation patterns to build distinct and reliable factors, and (2) Bartlett's test of sphericity, which represented whether the correlation matrix is significantly different from an identity matrix. The result of KMO (0.794) and Bartlett's *χ*^2^ value (2620.061, *p* < 0.001) in this study met the conditions for continuing the exploratory factor analysis (EFA). The diagonal of the antiimage correlation matrix is over 0.5.

There were three factors retained based on the eigenvalue >1 rule, and the same numbers of factors are also shown based on scree plot. The principal axis factoring was used, and the initial eigenvalues showed 86.72% of the total cumulative variance. The first factor is 38.68%, the second is 32.16%, and the third is 15.88%. The factor-loading matrix for the final is presented in Table 2. Most items corresponded with the original subscale, except for perceived benefit 5. Based on the factor analysis calculation, three factors emerged from the data set and had eigenvalues over Kaiser's criterion of 1.

**Figure 1:**
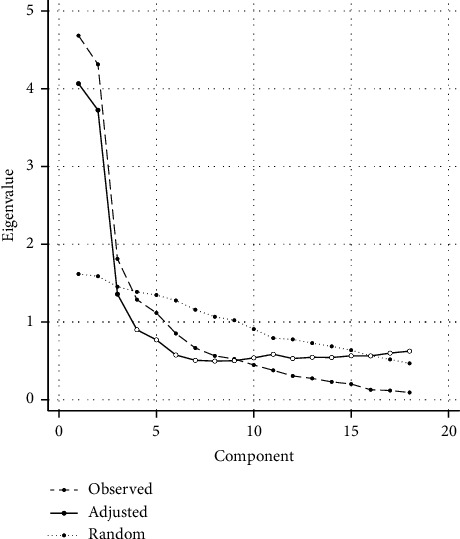
The comparison of scree plot and simulated parallel analysis within 95%.

The confirmation of sufficient factors was determined from the Scree plot and a parallel analysis (PA) (see Figure 1). PA was based on the calculation of randomly generated multiple data matrices, which have the same number of variables and cases as the original raw data set. Subsequently, differences between randomly and empirically generated eigenvalues are tested, and a significantly higher random dataset eigenvalue indicates the cutoff point for true factor numbers. After conducting PA (principal axis/common factor analysis, 95%), we retained three factors emerging through EFA, as shown in the PA result (Figure 1). However, item 5 of perceived benefit dimension was not in the same factor as the originals.

The difficulty index of the overall knowledge subscale was 0.856. Most of the items were easy for respondents to answer. The most difficult items were Knowledge 8, and the easiest was Knowledge 2 (Table 3).

**Figure 2:**
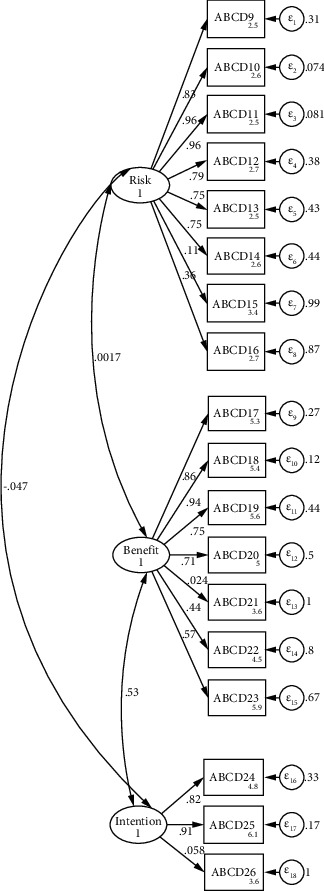
CFA model for original ABCD Risk Questionnaire.

**Figure 3:**
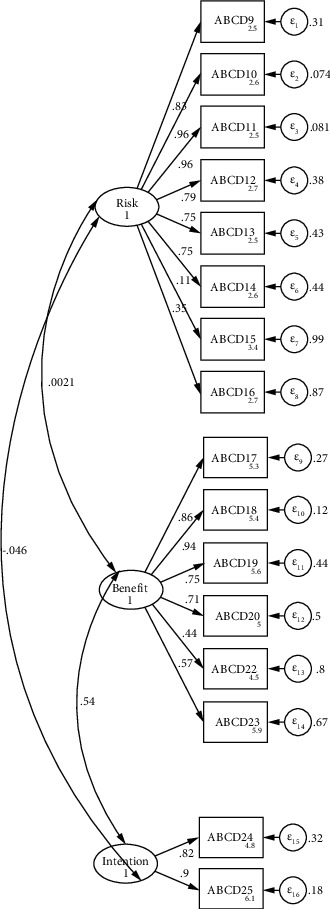
CFA model for ABCD Risk Questionnaire with 2 items taken.

The CFA was done to prove the structure using the new data from Indonesia. We tested two models of CFA; the first model included 18 items of ABCD Risk Questionnaire (Figure 2), whereas the second model included only 16 items (item numbers 21 and 26 were taken out) (Figure 3). The internal loading of the first model showed reliability ranging from 0.05 to 0.96. The goodness of fit result indicated from first CFA was not good (*χ*^2^ = 424.74, df = 132, *p* < 0.001, SRMR = 0.102, RMSEA = 0.138, CFI = 0.792, and TLI = 0.759). On the other hand, the internal loading of the second model showed reliability ranging 0.11–0.96, whereas the goodness model of fit was acceptable good (*χ*^2^ = 333.81, df = 101, *p* < 0.001, SRMR = 0.081, RMSEA = 0.140, CFI = 0.827, and TLI = 0.795).

### 3.4. The Reliability of the ABCD-Indonesian Version

Cronbach's alpha (*α*) is commonly used to evaluate a questionnaire's internal consistency/reliability. This study showed that the Cronbach's (*α*) of the entire questionnaire was 0.737; for each domain sequentially, it was 0.462, 0.873, 0.787, and 0.431. In consideration of the ordinal scales used in the instrument (question 9 to question 26), we also calculated the ordinal alpha [24, 25]. The result showed that the ordinal alpha of the entire questionnaire was 0.638, whereas each factor sequentially showed the ordinal alpha of 0.859, 0.828, and 0.676 (Table 4).

## 4. Discussion

In this study, we adopted the Indonesian ABCD Risk Questionnaire with an online survey of adult respondents. The original questionnaire consists of the following four domains: CVDs knowledge, perceived risk of heart attack/stroke, perceived benefits, and intentions to change, and healthy eating intentions [9]. The content validity of our findings shows the overall I-CVI and S-CVI above the expected (0.78 and 0.70), which means the Indonesian version of ABCD risk is valid [26]. The two items with an I-CVI under 0.70 are modified to be more understandable in Indonesian. Item number 8, “a family history of heart disease is not a risk factor for high blood pressure,” is a negative statement, and it is a common statement in the Indonesian population. The respondents may misunderstand whether the answer is true or false since this statement might confuse the reader. It is also shown in the difficulty index that item number 8 has the lowest grade. The difficulty index of 0.7 is considered easy to answer by respondents [27]. This item was changed into a positive statement: “*a family history of heart disease is a risk factor for high blood pressure*”. Item number 9, “I feel I will suffer from a heart attack or stroke sometime during my life” is too forthright, and the respondents are confused about how to respond. Asian people, including Indonesians, are more likely to choose fewer extreme responses [28]. We changed item number 9 to “*there is a possibility that I will have a heart attack or stroke*” as the initial development by Woringer et al. [9].

The unidimensional test using the Mokken analysis showed that a coefficient of scalability (H) reflected the result of *H* ≥ 0.5 [29]. All 18 items constructed in the questionnaire showed that H ranged between 0.910 and 0.409 (all are above 0.4) [30]. This result implies that the scalability of the questionnaire is good and then all the questions were retained as a set of questionnaires. Furthermore, this study showed the KMO result of 0.79 with a significant *p* value of Bartlett's Test of Sphericity (0.0001), indicating the sample size was adequate for EFA analysis. A KMO level of 0.5 is suitable for analysis, whereas 0.8 is best for analysis [31]. On the other hand, the *p* value significance of Bartlett's test of sphericity reflects that the analysis could proceed [17]. The goodness of fit of the first CFA model of CFA was not satisfactory. However, the second model showed a better and more acceptable model compared to the first model (reflected through higher TLI and CFI values and SRMR value of 0.08 considered as high). The SRMR provides the accuracy test of fit better than RMSEA [32].

The EFA and CFA showed that Indonesia ABCD questionnaire has three latent variables. In our findings, three factors emerged based on eigenvalues (>1) of factor analysis. Following this, we conducted parallel analysis (PA) to determine the appropriate number of factors (see Figure 1). PA calculates the eigenvalues of randomly generated multiple data matrices with the same number of variables and cases as the original raw dataset. The differences between randomly generated and empirical eigenvalues were then tested. The cutoff point of this comparison will extract the retained factors. Therefore, three factors were retained through the EFA and PA analysis. This finding shows a similar result to the original questionnaire, as well as other versions from several different countries (China: 3 factors, Hungary: 3 factors, United Kingdom: 3 factors, and Netherlands: 3 factors [12–14, 33]). However, based on EFA result in this study, item 5 of perceived benefit factor will be allocated into different factors from the original instrument.

Based on CFA, practically all ABCD items had a three-factor structure, which was similar to the original instruments. However, as items 21 and 26 had low factor loading scores, these were removed from the model to improve the model's goodness of fit. These two questions were negative remarks on the ABCD Risk Questionnaire. There is a lack of research on negative statements in Likert scales in Indonesia; nonetheless, previous research suggests that both positive and negative statements should not be included in a scale [34]. The presence of these two types of statements caused confusion for the respondent [34]. Therefore, we recommend changing the negative statements into positive ones.

The internal consistency of the overall scale was lower than the original scale (0.85, 0.82, and 0.56 for perception risk, benefit, and healthy eating intentions) [9]. Even though Cronbach's *α* for two domains (knowledge and intention to change) were below the minimum threshold of 0.70, we found that the entire questionnaire's reliability was strong as a set. Furthermore, considering the Likert scale used in the instrument (from question 9 to 26), we also calculated the ordinal *α* for the entire instrument (0.638) that showing a lower *α* score than the Cronbach *α* (0.737). However, almost all the ordinal *α* for each factor (assessing with ordinal scale) were greater than the Cronbach *α*. Therefore, considering the factor analysis study using the Likert-style scale, we are more confident on the reliability result calculated with ordinal coefficient alpha [25]. Our findings were similar to the Malay version that internal consistency of that version was over 0.70 [14]. Furthermore, the low Cronbach's *α* was acceptable as the original questionnaire for domain intention to change because this domain only has a three-item scale.

## 5. Limitation

This study was distributed through an online platform. Consequently, the demographic data do not vary, especially on religion and background education. However, the number of respondents was significant to achieve the data variation. Moreover, there are several Indonesian terminologies which could not be as precise as the meaning of English terminologies. Therefore, we considered the panelist' difficulties in selecting the most representative Indonesian terminologies during the forward translation. This issue may lead to ambiguity among the respondents in interpreting the questions and statements in the questionnaire.

## 6. Conclusion

Based on psychometric investigation, this study concluded that the Indonesian version of ABCD questionnaire is a valid and accurate to be used for assessing the knowledge, perceived risk, perceived benefit, and intention to change. A few items on the ABCD questionnaire have been modified to be fitted into Indonesian cultural norms and make the questionnaire easier to respond. Because CVDs are common in low-income countries, this conclusion can be extended to the original study to assess Indonesian attitudes and beliefs.

## Figures and Tables

**Table 1:** Sociodemographic characteristics of the respondents (*N* = 236).

Variables	*n* (%)	Mean (SD)	Range
Age		26.41 (5.252)	18–44
Gender			
Male	91 (38.6)		
Female	145 (61.4)		
Education			
Secondary	1 (0.4)		
High school	75 (31.8)		
University	160 (67.8)		
Religion			
Muslim	75 (31.8)		
Christian Protestant	137 (58.1)		
Catholic	17 (7.2)		
Buddha	5 (2.1)		
Confucian	2 (0.8)		
Occupation			
Unemployed, fulltime mom	22 (9.3)		
Student	54 (22.9)		
Freelance	11 (4.7)		
Government	16 (6.8)		
Private	116 (49.2)		
Self-employed	17 (7.2)		
Income			
No income	59 (25.0)		
<IDR 1.000.000	17 (7.2)		
IDR 1.000.000–3.000.000	34 (14.4)		
IDR 3.000.000–5.000.000	43 (18.2)		
>IDR 5.000.000	83 (35.2)		
Smoking habit			
Smoking	45 (19.1)	Length (in month(s))	Length (in month(s))
Not smoking	191 (80.9)	14.82 (42.24)	1–240
Body Mass Index classification			
Underweight	19 (8.1)		
Normal	76 (32.2)		
Overweight	38 (16.1)		
Obesity 1	64 (27.1)		
Obesity 2	34 (14.4)		
Daily activities			
No exercise	64 (27.1)		
Mild exercise	13 (5.5)		
Moderate exercise	108 (45.8)		
Heavy exercise	51 (21.6)		
Awareness (80 marks)		55.24 (5.837)	34–76
Knowledge (8 marks)		6.89 (1.159)	2–8
Perceived risk (32 marks)		17.29 (4.621)	8–32
Perceived benefits (28 marks)		22.27 (2.878)	10–28
Intention to change (12 marks)		8.80 (1.408)	5–12

**Table 2:** Factor loadings (FLs) of EFA.

Nos.	Item	Factors			Communality	Uniqueness
		1	2	3		
1	Perceived risk 1	0.84			0.72	0.28
2	Perceived risk 2	0.83			0.74	0.26
3	Perceived risk 3	0.85			0.79	0.21
4	Perceived risk 4	0.80			0.69	0.31
5	Perceived risk 5	0.79			0.66	0.34
6	Perceived risk 6	0.75			0.61	0.39
7	Perceived risk 7	0.10			0.09	0.91
8	Perceived risk 8	0.41			0.20	0.80
9	Perceived benefit 1		0.81		0.72	0.28
10	Perceived benefit 2		0.77		0.71	0.29
11	Perceived benefit 3		0.76		0.66	0.34
12	Perceived benefit 4		0.68		0.50	0.50
13	Perceived benefit 5			0.56	0.36	0.64
14	Perceived benefit 6		0.48		0.28	0.72
15	Perceived benefit 7		0.77		0.64	0.36
16	Intention to change 1			−0.54	0.62	0.38
17	Intention to change 2			−0.58	0.69	0.31
18	Intention to change 3			−0.34	0.23	0.77

*Note.* The “minimum residual” extraction method was used in combination with “Varimax” rotation; the hidden loadings were below 0.3.

**Table 3:** Item statistics of knowledge subscale of ABCD.

Nos.	Item	Difficulty index
1	Knowledge 1	0.915
2	Knowledge 2	0.970
3	Knowledge 3	0.936
4	Knowledge 4	0.831
5	Knowledge 5	0.983
6	Knowledge 6	0.890
7	Knowledge 7	0.852
8	Knowledge 8	0.492

**Table 4:** Reliability estimated with Cronbach *α* and ordinal *α*.

Factor	Cronbach	Ordinal
Perceived risk	0.873	0.859
Perceived benefits	0.787	0.828
Intention to change	0.431	0.676
Overall questionnaire	0.737	0.638

## Data Availability

The survey's raw data will not be available to protect the personal information of the study responders.
